# Force plate vertical jump scans are not a valid proxy for physical fitness in US special warfare trainees

**DOI:** 10.3389/fphys.2022.966970

**Published:** 2022-11-16

**Authors:** W. Casan Scott, Ben R. Hando, Cody R. Butler, John D. Mata, Jacob F. Bryant, Siddhartha S. Angadi

**Affiliations:** ^1^ US Air Force Special Warfare Training Wing, San Antonio, TX, United States; ^2^ National Council on Compensation Insurance, Boca Raton, FL, United States; ^3^ Kennell & Associates, Inc., Fall Church, VA, United States; ^4^ Department of Kinesiology, School of Education and Human Development, University of Virginia, Charlottesville, VA, United States

**Keywords:** tactical, performance, testing, military, SOF, KPI

## Abstract

**Background:** The United States Air Force Special Warfare Training Wing (SWTW) administers a comprehensive physical fitness test to active duty Airmen entering the Special Warfare training pipeline. The Sparta Science™ system utilizes proprietary software to analyze the force-time curve of a vertical jump and purports to serve as a proxy for traditional military fitness tests. The Sparta Science™ system produces four proprietary metrics, including the Sparta™ Score, which is correlated to high magnitudes of force production purportedly *performance.* This study investigated how Sparta™ Jump Scans correlate to components of a physical fitness test utilized within the SW training pipeline.

**Methods:** At the entry and exit of an 8-week Special Warfare Training Wing preparatory course (SW PREP), 643 trainees completed both an initial and final Sparta™ Jump Scan and a Candidate Fitness Test (CFT). The Candidate Fitness Test consists of eight components and tests several different domains of fitness including strength, power, muscular endurance, swimming proficiency, and cardiovascular fitness. Paired t-tests were used to determine if Sparta™ Jump Scan metrics and CFT components changed during SW PREP. Sparta™ Score’s correlation was assessed against every other Sparta™ Jump Scan metric and all CFT fitness measures.

**Results:** This study found that the Sparta™ Jump Scan metrics decline slightly over SW PREP (*p* < 0.05; negligible-small effect size), while most CFT measures improve (*p* < 0.05; small-medium effect size). Changes in Sparta™ Jump Scan metrics did not reflect the changes in CFT performance over SW PREP (*r* ^2^: 0.00–0.03).

**Conclusion:** The Sparta™ Score was not correlated to the most tactically-relevant fitness measures (rucking and swimming), and only weakly correlated with the only jumping measure on the fitness test, the standing broad jump.

## Introduction

The United States Air Force (USAF) Special Warfare (SW) career field requires high levels of physical abilities across several domains, including aerobic and anaerobic fitness, muscular strength, muscular endurance, coordination, and others (Pearce, 2016; Robson et al., 2018). Military occupations are physically demanding, but particularly so for Special Operations Forces (SOF) (Grier et al., 2018; Royer et al., 2018; Stannard and Fortington, 2021). Optimizing physical fitness benefits SOF units by improving performance, productivity, and overall well-being, while reducing injuries and lost workdays (Knapik et al., 1993; Skeehan et al., 2009; Knapik and East, 2014; Keenan et al., 2017; Grier et al., 2018; Royer et al., 2018; Feickert, 2021; Stannard and Fortington, 2021). Fitness assessments enable military leadership to track physical fitness levels, enforce physical fitness standards, determine the effectiveness of training regimens, and identify unit and individual weaknesses (Knapik et al., 1993; Knapik and East, 2014; Pearce, 2016; Keenan et al., 2017; Grier et al., 2018; Robson et al., 2018; Royer et al., 2018). The Candidate Fitness Test (CFT) was developed by the USAF to reflect a broad set of physical fitness attributes that are based on USAF SW mission-specific physical duty requirements (Pearce, 2016; Robson et al., 2018; Tier two Operator Physical Fitness Tests and Standards for Special Warfare Operators (CCT, PJ, SOWT, STO & CRO) Test Guidance, 2019). Since 2019, the USAF Special Warfare Training Wing (SWTW) has administered the CFT to active duty Airmen entering the SW training pipeline (Pearce, 2016; Robson et al., 2018; Tier two Operator Physical Fitness Tests and Standards for Special Warfare Operators (CCT, PJ, SOWT, STO & CRO) Test Guidance, 2019). The CFT consists of eight distinct tests (see methods) and requires substantial time (i.e., 4–8 h) and specialized equipment (i.e., bars, weights, cones, pool, a 60 lb rucksack, *etc.*) for administration (Tier two Operator Physical Fitness Tests and Standards for Special Warfare Operators (CCT, PJ, SOWT, STO & CRO) Test Guidance, 2019). Because of this, human performance teams working in formal training environments often seek key performance indicators to serve as a proxy for comprehensive fitness assessments (Passos et al., 2021). For instance, the countermovement jump (CMJ; i.e., maximal effort vertical jumps) is frequently implemented as a key performance indicator of lower body power output, readiness-to-train, and training fatigue (Claudino et al., 2017). CMJ height is traditionally measured by the increase in vertical reach at the apex of a CMJ, or through an estimation based on time-of-flight or change in momentum using a force-plate (Claudino et al., 2017). With force-plate technology, additional kinetic measurements can be recorded during a CMJ such as power, force, velocity, and impulse (Claudino et al., 2017). Sparta Science™ (Menlo Park, CA) produces a commercially available force-plate technology system that estimates vertical jump measurements and assesses movement and overall fitness levels through repeated CMJs (Wagner and Frost, 2020). Data from repeated CMJs (4–6 successive jumps are recommended by Sparta Science ™) are captured by the Sparta Science ™ force-plates and converted into proprietary scores that, per the company’s instructions, may be used to inform strength and conditioning programs (Sparta Jump Scan 101: Load, Explode, and Drive; Wagner and Frost, 2020).

The Department of Defense (DoD) has invested heavily in force-plate technologies, including the Sparta Science™ system (William M. (Mac) Thornberry National Defense Authorization Act for Fiscal Year 2021: Report of the Committee on Armed Services House of Representatives on H.R. 6395, 9 July 2020; Manfre, 2020). Units in all four major branches of the military are currently using force-plate technology to assess “unit readiness” and make training programming decisions (William M. (Mac) Thornberry National Defense Authorization Act for Fiscal Year 2021: Report of the Committee on Armed Services House of Representatives on H.R. 6,395, 9 July 2020; Manfre, 2020). Specifically, the Sparta Science™ system claims to help many of these military units devise custom fitness programs and provides these units with automated recommendations for physical training adjustments (Report on Force Plate Technology Utilizing Machine Learning for Improving Combat Readiness, 2022). Sparta Science™ claims, “[r]ather than the current annual physical fitness tests, service members can be scanned weekly or monthly, giving leaders the ability to hold individuals accountable for progress and a clearer, up-to-date appraisal of overall fitness levels” (Wagner and Frost, 2020). However, despite these claims and the widespread use of the Sparta Science™ system within the DoD, there are currently, no published, peer-reviewed studies demonstrating that this force-plate system is a valid proxy for military physical fitness. Thus, this study aims to determine (1) how changes in Sparta™ Jump Scans compare to changes in various physical fitness measures of USAF Airmen during an 8-week SW preparatory course (SW PREP), and (2) evaluate if Sparta™ Jump Scans are correlated to physical fitness measures utilized within the SWTW pipeline.

## Methods

### Participants

**FIGURE 1:**
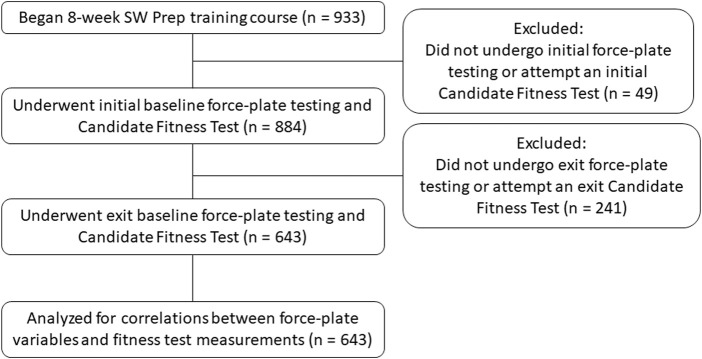
Flowchart accounting for subject participation and those lost to follow up. SW Prep = Special Warfare Preparatory Course.

The 59th Medical Wing’s Institutional Review Board approved this study protocol. This study was conducted at the USAF SWTW at Joint Base San Antonio, TX. Subjects were USAF Airmen entering the SW training pipeline through an 8-week preparatory course (SW PREP) designed to prepare Airmen for the rigors of the SW training pipeline, which has a high rate of attrition and MSKIs (musculoskeletal injuries) (Hando et al., 2021). SW trainees take the CFT and Sparta™ Jump Scan at two time points: (1) within 3 days prior to entering SW PREP (referred to as *Initial* measures in this study), and (2) within 3 days of the final day of the SW PREP (referred to as *Final* measures in this study). From October 2019 through March 2021, 977 male trainees entered SW PREP, of which 643 completed both an initial and final Sparta™ Jump Scan and CFT at the entry and exit of SW PREP, respectively. Data collection of the Sparta™ Jump Scan metrics and CFT is a component of normal operations for all USAF SWTW trainees, and informed consent is obtained from all trainees prior to beginning the training pipeline. Subject participation is summarized in Figure 1 and trainee physical characteristics are described in Table 1.

**TABLE 1:** Baseline demographic information, fitness, and Sparta TM values for Air Force Special Warfare (SW) trainees entering and exiting the SW Preparatory Course (n = 643). Initial measurements are collected upon entry of the SW Preparatory Course, and Final measurements are collected upon exit from the SW Preparatory Course.

	Mean (SD)	
Static Measures		
Age (years)	22.1 (3.9)	
Height (cm)	1.78 (0.06)	
	**Initial**	**Final**
Body Mass		
BMI (kg/m2)	25.3 (2.1)	25.4 (2.1)
Mass (kg)	79.7 (8.5)	80.1 (8.6)
Fitness		
Broad Jump (cm)	247.7 (18.8)	246.4 (17.8)
Left Agility Drill (seconds)	5.03 (0.33)	4.95 (0.24)
Right Agility Drill (seconds)	4.95 (0.24)	4.95 (0.25)
Max Deadlift (kg)	149.0 (20.2)	155.3 (17.7)
Pullups	13.5 (3.5)	13.8 (3.4)
100 yard Farmer’s Carry (seconds)	20.9 (2.3)	20.2 (2.1)
300 yard Shuttle (seconds)	66.6 (3.0)	67.3 (3.3)
1,500 m Fin (mins secs)	34 m 46 s (4 m 8 s)	32 m 32 s (3 m 34 s)
3 mile Ruck (mins secs)	40 m 44 s (3 m 22 s)	38 m 27 s (4 m 6 s)
Sparta Jump Scan		
Sparta Score	81.2 (3.7)	80.3 (3.4)
Load	48.7 (9.3)	47.5 (7.7)
Explode	43.8 (7.5)	42.3 (7.1)
Drive	52.5 (8)	50.0 (7.8)
Vertical Jump Height (cm)	41.9 (5.8)	39.1 (5.3)

Data are presented as mean and standard deviations (SD).

### Design and setting

Upon entry into SW PREP, all trainees undergo routine baseline testing, to include examinations by sports medicine staff, to screen for existing injuries that would disqualify them from training. Trainees with active injuries were removed from training and not included in this analysis. Data were collected from seven cohorts of the 8-week SW PREP course from October 2019 through March 2021.

The Sparta Science™ system utilizes software to analyze the force-time curve of a vertical jump, and produces four proprietary metrics: *Sparta™ Score, Load, Explode, and Drive*. The Sparta™ Score is correlated to high magnitudes of force production and a balanced ratio between the Load, Explode, and Drive scores (Sparta Jump Scan 101: Load, Explode, and Drive). The Load Score is the average eccentric rate of force development, and the Explode score is the average concentric rate of force development. The Load and Explode scores are normalized to data within a Sparta Science*™*-maintained database (Sparta Jump Scan 101: Load, Explode, and Drive). The Drive score represents a normalized value for impulse, which is a measure of both the magnitude and duration of force (Sparta Jump Scan 101: Load, Explode, and Drive). Additionally, the Sparta™ Jump Scans also record body weight and vertical jump height (these measures are used in this study).

The CFT consists of eight events: standing broad jump, 5-10-5 m agility drill, trap bar deadlift, pullups, 100 yard farmer’s carry, 300 yard shuttle run, 1,500 m finned swim (a swimming event in which trainees are fitted with fins), and a three mile ruck March (a three mile timed March while carrying a 60 pound rucksack) (Tier two Operator Physical Fitness Tests and Standards for Special Warfare Operators (CCT, PJ, SOWT, STO & CRO) Test Guidance, 2019). The CFT aims to measure the physical abilities necessary for SW operators to perform the critical physical tasks inherent to their operational duties (Tier two Operator Physical Fitness Tests and Standards for Special Warfare Operators (CCT, PJ, SOWT, STO & CRO) Test Guidance, 2019). The standing broad jump is a measure of lower body explosive power, and is relevant to power generation in a tactical environment. In the 5-10-5 yard agility drill, a trainee assumes a three-point stance straddling a starting point. The trainee begins by sprinting 5 yards, touching a line, reversing direction, sprinting 10 yards and touching another line, again reversing direction, and finally sprinting a final 5 yards and ending where the drill initially began. The 5-10-5 agility drills tests in both the left and right directions, and aims to measure agility, coordination, and reaction time. The tactical relevance of the 5-10-5 agility drills is rapid acceleration, change of direction, and mobility. The deadlift max lift utilizes a “trap bar”, and measures lower body muscular strength. The tactical relevance of the deadlift is the ability to lift and move heavy equipment or personnel. Pull-ups measure upper body muscular endurance and are relevant to infiltration/exfiltration tactical actions requiring vertical movement. In the 100 yard Farmer’s Carry, trainees carry two 53 pound kettlebells for 100 yards as quickly as possible. The Farmer’s Carry measures anaerobic capacity and grip strength, and is relevant for tactical movements requiring operators to carry equipment or personnel. In the 300 yard shuttle, trainees run 12 legs of 25 yards, or six round trips measuring 50 yards each in length. The 300 yard shuttle aims to measure both anaerobic and aerobic capacity. The 1,500 m finned swim (i.e., 1,500 m fin) measures cardio-respiratory endurance and combat swimmer skill. The testing purpose of the three mile ruck is to measure cardio-respiratory endurance, and the tactical relevance is tactical infiltration and dismounted operations requiring load carriage skills (Tier two Operator Physical Fitness Tests and Standards for Special Warfare Operators (CCT, PJ, SOWT, STO & CRO) Test Guidance, 2019).

### Statistical analysis

Descriptive statistics were calculated for age, height, body weight, BMI, and each CFT and Sparta™ jump scan measure during the Initial and Final testing time-points. A paired samples *t*-test and Cohen’s D was used to determine whether Sparta™ Jump Scan metrics and CFT fitness measurements changed significantly between the beginning and end of SW PREP (Field et al., 2012). If a comparison of mean differences violated the assumption of equal variance, variance was pooled using Satterthwaite’s approximation of the degrees of freedom (Satterthwaite, 1941). Because the distribution of Max Deadlift differences deviated significantly from a normal distribution, the difference in Max Deadlift means was determined using the Wilcoxon Signed-Ranks test, (Field et al., 2012), and the effect size was estimated according to recommendations by Rosenthal and Rubin (Eq. 1) (Rosenthal and Rubin, 1991).

$$r=z÷\sqrt{N.}$$ (1)

To investigate the correlation between Sparta™ Score and CFT measures, we calculated the Sparta™ Score’s correlation coefficients (*r*) and coefficients of determination (*r* ^*2*^) against all CFT fitness measures. To better understand how the Sparta™ Jump Scan metrics were related, we also investigated the correlation and shared variance (*r and r* ^*2*^) between the Sparta™ Score and all other Sparta™ Jump Scan metrics. Sparta™ Score’s correlation to vertical jump height was of particular interest because there is a broader body of research on the vertical jump in tactical populations. Correlations were calculated first among Initial measurements, and later among Final measurements (i.e., Initial and Final measurements were not pooled). Additionally, correlations were also calculated between the delta Sparta™ Score (i.e., Final–Initial measurement) and the deltas of every other Sparta™ Jump Scan metric and the OFT fitness measures. Statistical analyses were conducted in *R* (R Development Core Team, 2007) and figures were produced using the package ggplot2 (Wickham, 2016). Effect size for paired-tests were calculated using the *effsize* package (Torchiano). *R* code used to calculate effect size for the Wilcoxon Signed-Rank test using Rosenthal’s formula was published in *Discovering Statistics using R* (Field et al., 2012).

## Results

Sample descriptive statistics, calculated by initial and final time points, are presented in Table 1. At the conclusion of SW PREP, there were significant differences (initial vs. final) in all Sparta™ Jump Scan metrics and CFT measurements (*p* < 0.05). SW Trainees gained an average of 0.4 ± 2.1 kg body mass (*p* < 0.01, Cohen’s D = 0.05). All Sparta™ Jump Scan metrics decreased between the beginning (initial scores) and end (final scores) of SW PREP. At the end of SW PREP, Sparta™ Scores decreased, on average, by 0.9 points (95% Confidence Interval [CI] = −1.1, −0.7; *p* < 0.01; Cohen’s D = 0.26). Load, Explode, and Drive also decreased during SW PREP by 1.2 points (95% CI = −1.8, −0.7; *p* < 0.01; Cohen’s D = 0.15), 1.5 points (95% CI = −1.9, −1.1; *p* < 0.01; Cohen’s D = 0.21), and 2.5 (95% CI = −3.1, −2.0; *p* < 0.01; Cohen’s D = 0.32), respectively. Vertical jump height, which is moderately correlated with most Sparta™ Jump Scan metrics, decreased by 2.8 cm (95% CI = −3.0, −2.5; *p* < 0.01; Cohen’s D = 0.51), on average. Additionally, trainees increased their body weight by an average of 0.4 kg (95% CI = 0.3, 0.6; *p* < 0.01; Cohen’s D = 0.05).

**FIGURE 2:**
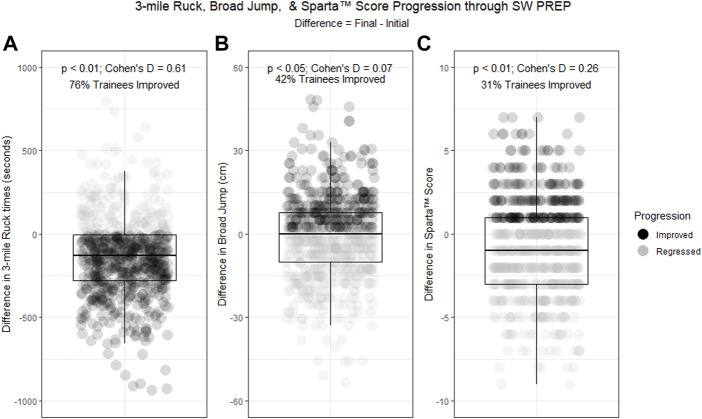
Box-and-whisker plots displaying the distribution of differences (Final–Initial) for **(A)** 3-mile Ruck (seconds), **(B)** Broad Jump (cm), and **(C)** Sparta™ Scores for SW Prep trainees. A paired *t*-test was used to compare mean differences against the null. The *p*-value and Cohen’s D are reported for each paired comparison. Each point represents an individual trainee’s measure of difference (Final–Initial) in that event. Trainees who improved their score for that event during SW Prep are colored black, and the proportion of the cohort that improved scores during SW Prep are annotated within each plot.

While the Sparta™ Jump Scan metrics decreased after the 8 weeks of SW PREP, most fitness measurements on the CFT improved. One exception, the broad jump, declined by an average of 1.27 cm (95% CI = −2.54, −0.51; *p* = 0.04; Cohen’s D = 0.07). Trainees improved, on average, both left and right 5-10-5 agility drills by 0.08 s (95% CI = −0.10, −0.06; *p* < 0.01; Cohen’s D = 0.27) and 0.10 s (95% CI = −0.13, −0.08; *p* < 0.01; Cohen’s D = 0.34), respectively. Maximum Deadlift increased by 6.4 kg (95% CI = 5.4, 7.3; *p* < 0.01; effect size = 0.41) and pullups increased by 0.3 reps (95% CI = 0.1, 0.5; *p* < 0.01; Cohen’s D = 0.09). Trainees improved their 100 yard Farmer’s Carry time by 0.7 s (95% CI = −0.9, −0.6; *p* < 0.01; Cohen’s D = 0.34). On average, trainees performed slower on the final 300 yard shuttle (mean difference = 0.7 s; 95% CI = 0.4, 0.9; *p* < 0.01; Cohen’s D = 0.21). Conversely, trainees improved both their 1,500 m fin and three mile ruck times by an average of 2 min 40 s (95% CI = −3 m 2s, −2 m 19s; *p* < 0.01; Cohen’s D = 0.59) and 2 min 17 s (95% CI = −2 m 36s, −1 m 58 s; *p* < 0.01; Cohen’s D = 0.61), respectively. Results from the paired t-tests are presented in Table 2. Overall, Sparta™ Jump Scan metrics depicted trends opposite of every CFT component except the broad jump (Table 2; Figure 2).

**TABLE 2:** Hypothesis testing results for comparisons of initial vs. final means of SpartaTM Jump Scan metrics and Candidate Fitness Test measurements. For all comparisons, except Max Deadlift, a paired *t*-test was used to test mean differences, and Cohen’s D was calculated to measure effect size. For Max Deadlift, mean differences were tested using a Wilcoxon Signed-Ranks test, and effect size was calculated according to recommendations by Rosenthal and Rubin (1991).

Fitness	Mean difference (95% CI)^a^	*p*-value	Effect size	Relative effect size
Broad Jump (cm)	−1.27 (−2.54, −0.51)	0.04	0.07	negligible
Left Agility Drill (seconds)	−0.08 (−0.10, −0.06)	<0.01	0.27	small
Right Agility Drill (seconds)	−0.10 (−0.13, −0.08)	<0.01	0.34	small
Max Deadlift (kg)	6.4 (5.4, 7.3)	<0.01	0.41^b^	small
Pullups	0.3 (0.1, 0.5)	<0.01	0.09	negligible
100 yd Farmer’s Carry (seconds)	−0.8 (−0.9, −0.6)	<0.01	0.34	small
300 yard Shuttle (seconds)	0.7 (0.4, 0.9)	<0.01	0.21	small
1500 m Fin (seconds)	−2 m 41s (−3 m 2s, −2 m 19s)	<0.01	0.59	medium
3 mile Ruck (seconds)	−2 m 17s (−2 m 36s, −1 m 58s)	<0.01	0.61	medium
Sparta™ Jump Scan				
Sparta Score	−0.9 (−1.1, −0.7)	<0.01	0.26	small
Load	−1.2 (−1.8, −0.7)	<0.01	0.15	negligible
Explode	−1.5 (−1.9, −1.1)	<0.01	0.21	small
Drive	−2.5 (−3.1, −2.0)	<0.01	0.32	small
Vertical Jump Height (cm)	−2.8 (−3.0, −2.5)	<0.01	0.51	medium
Mass (kg)	0.4 (0.3, 0.6)	<0.01	0.05	negligible

^a^ Mean Difference = Final value–Initial Value.

^b^ Effect size was calculated using r = *z*/√N, as recommended by Rosenthal and Rubin. (Rosenthal and Rubin, 1991) R code used to calculate Rosenthal’s formula was published in *Discovering Statistics using R*. (Field et al., 2012).

Initial and Final Sparta™ Scores demonstrated a stronger correlation with the other Sparta™ Jump Scan metrics (excluding *Drive*), compared to CFT measurements (Table 3). Sparta™ Scores were most strongly correlated with Vertical Jump Heights (*r*_*initial*_ = 0.73*, r*_*final*_ = 0.71; *r*_*initial*_^*2*^ = 0.53*, r*_*final*_^*2*^ = 0.50). Load, Explode, and Drive displayed weak to moderate correlations to Sparta™ Score, but body weight displayed nearly no correlation to Sparta™ Scores (*r*_*initial*_ = 0.07*, r*_*final*_ = 0.04; *r*_*initial*_^*2*^ = 0.00*, r*_*final*_^*2*^ = 0.00). Correlations between Sparta™ Score and CFT measurements were weak to non-existent. Among CFT events, Sparta™ Score was most strongly correlated to standing broad jump (*r*_*initial*_ = 0.45*, r*_*final*_ = 0.47; *r*_*initial*_^*2*^ = 0.20*, r*_*final*_^*2*^ = 0.22). However, Sparta™ Score explained very little variance in any other CFT event, as only four events (broad jump, left 5-10-5 agility drill, 100 yard farmer’s carry, and maximum deadlift) displayed an *r* ^*2*^ exceeding 0.10 (10% variance explained). Importantly, Sparta™ Score displayed no correlation to the most tactically relevant events, the 1,500 m fin (*r*_*initial*_ = −0.02*, r*_*final*_ = 0.01; *r*_*initial*_^*2*^ = 0.00*, r*_*final*_^*2*^ = 0.00) and three mile Ruck (*r*_*initial*_ = −0.06*, r*_*final*_ = −0.08; *r*_*initial*_^*2*^ = 0.00*, r*_*final*_^*2*^ = 0.01). Delta Sparta™ Scores demonstrated no correlation to the delta of any CFT measurement (Table 3).

**TABLE 3:** Correlation coefficients (*r*) and coefficients of determination (*r* ^*2*^) for individual correlations between the Sparta TM Score and (A) Sparta TM Jump Scan metrics, and (B) Candidate Fitness Test measures.

	Sparta score					
	Initial		Final		Δ (Final - Initial)	
	r	*r* ^2^	r	*r* ^2^	r	*r* ^2^
Sparta Jump Scan						
Load	0.57	0.32	0.52	0.27	0.55	0.30
Explode	0.66	0.44	0.61	0.37	0.51	0.26
Drive	−0.17	0.03	−0.15	0.02	−0.24	0.06
Vertical Jump Height	0.73	0.53	0.71	0.50	0.49	0.24
Body Mass	0.07	0.00	0.04	0.00	−0.04	0.00
Fitness						
Broad Jump	0.45	0.20	0.47	0.22	0.14	0.02
Left Agility Drill	−0.34	0.12	−0.32	0.10	−0.15	0.02
Right Agility Drill	−0.30	0.09	−0.29	0.08	−0.14	0.02
Max Deadlift	0.34	0.12	0.30	0.09	0.17	0.03
Pullups	0.27	0.07	0.25	0.06	0.09	0.01
100 yard Farmer’s Carry	−0.32	0.10	−0.36	0.13	−0.08	0.01
300 yard Shuttle	−0.20	0.04	−0.06	0.00	0.13	0.02
1500 m Fin	−0.02	0.00	0.01	0.00	−0.07	0.00
3 mile Ruck	−0.06	0.00	−0.08	0.01	0.03	0.00

## Discussion

The main findings of this study were that (1) the Sparta™ Score does not reflect change in a USAF Airman’s physical fitness during an 8-week Special Warfare preparatory course (SW PREP), and (2) the Sparta™ Score was not correlated to comprehensive physical fitness as captured by current fitness tests within the USAF SW training pipeline. Further, the Sparta™ Score was not correlated to the most tactically-relevant fitness measures (i.e., rucking under external load and swimming), and only weakly correlated with the only jumping measure on the CFT, the standing broad jump. This is unsurprising given the principle of specificity (Swain et al., 2012). However, in light of persistent vendor claims, it remained to be tested. A primary goal of the SW training pipeline, and specifically SW PREP, is to improve the physical fitness level of SW candidates in order to meet pre-specified fitness benchmarks that are assessed throughout the training pipeline and during an SW operator’s career. Periodic fitness testing informs staff and coaches of each candidate’s physical deficiencies, and allows them to adjust training where needed to help candidates meet these fitness testing benchmarks. It is therefore critical that SW staff and coaches have accurate appraisals of each candidate’s longitudinal fitness trends during a given training period. Using metrics that do not reflect relevant fitness levels can mislead coaches and staff, misinform training plans, and compromise the physical development of SW candidates. For example, because the Sparta™ Score does not account for variability in every non-power related fitness component, it fails to accurately capture USAF SW trainees’ fitness progression during SW PREP. For instance, 76% of trainees in this study improved their 3-mile ruck times during SW PREP, compared to 42% improving broad jumps and only 31% improving Sparta™ Scores (Figure 2). If USAF SW used the Sparta™ Score as a proxy for the CFT, they would inaccurately assess physical fitness progression for the majority of their population. Because Sparta™ Jump Scan metrics are the product of a vertical jump, they are unlikely to capture many other aspects of physical fitness beyond those strongly correlated to lower extremity power output. While there are no peer-reviewed studies examining Sparta™ Jump Scan metrics correlation to overall physical fitness, there is a wealth of evidence detailing the relationship between vertical jump and tactically-relevant measures of fitness. For instance, it has been previously established that the vertical jump alone is very weakly correlated (r = -0.14; *r* ^2^ = 0.02) to road marches, which are among the most tactically-relevant fitness measures for military personnel (Knapik et al., 1990). Recent research on US Army Combat Arms’ soldiers revealed that the standing long jump (e.g., broad jump) had essentially no correlation to a series of occupationally related tests which included a tactical March, movement under fire, a sandbag carry, and a casualty drag (Boye et al., 2017; Foulis et al., 2017; Sharp et al., 2017). In addition to these observations from within the DoD, other research shows jumping ability is also not correlated with success in tactical Police (Schram et al., 2020) or the Australian Army Special Forces (Hunt et al., 2013). Results from the current study clearly support previous findings that the vertical jump, or vertical jump-derived metrics such as the Sparta™ Score, cannot be used as a surrogate measure of overall physical fitness in tactical populations.

In an exceedingly complicated tactical environment, a vertical jump-based metric is unlikely to reflect the multi-faceted fitness profile necessary for military SOF occupations. Even in team sports that place a premium on lower body power, a vertical jump neglects to capture the breadth of physical attributes necessary to play that sport. For instance, previous research on basketball players shows that vertical jump is only loosely correlated with sprinting ability and not correlated with VO_2_ max (Stojanovic et al., 2012). In military and other tactical units, comprehensive fitness profiles are used to create specific strength and conditioning programs, develop return to active duty guidelines, and inform recruit selection (Hunt et al., 2013). There are a wide variety of measures and protocols used among elite tactical units, but most measure muscular strength, power, and aerobic capacity while also tailoring the test to cover the relevant spectrum of fitness demands for each tactical population (Maupin et al., 2018). Despite the widespread use of force-plate technology within the DoD, the vertical jump scans evaluated in this study were not helpful in evaluating overall fitness level in Special Warfare (SW) candidates. Additionally, these scans were not helpful in assessing changes in fitness levels.

## Conclusion

The vertical jump force-plate scans assessed in this large cohort study produced scores that did not correlate to the physical fitness of Air Force Special Warfare trainees. In fact, the scores only accounted for ≤3% of the variance observed in the physical fitness tests and are not a valid proxy for commonly used fitness metrics. Military organizations seeking rapid assessments of physical fitness should consider abbreviated protocols of the most tactically relevant fitness components. As Richard Feynman succinctly put it, “For a successful technology, reality must take precedence over public relations, for Nature cannot be fooled.”

## Strength and limitations

Strengths of this study include its large sample size, high follow-up rate, and the controlled environment provided by the 8-week SW Prep training course. This study was limited by not accounting for each subject’s training experience prior to joining the USAF. This limitation was mitigated by using paired-sample comparisons of Sparta™ Jump Scan metrics and fitness measurements.

## Data Availability

The raw data supporting the conclusions of this article will be made available by the authors, without undue reservation.
